# A Prospective Analysis of Meat Mutagens and Colorectal Cancer in the Nurses’ Health Study and Health Professionals Follow-up Study

**DOI:** 10.1289/EHP238

**Published:** 2016-04-22

**Authors:** Ngoan Tran Le, Fernanda Alessandra Silva Michels, Mingyang Song, Xuehong Zhang, Adam M. Bernstein, Edward L. Giovannucci, Charles S. Fuchs, Shuji Ogino, Andrew T. Chan, Rashmi Sinha, Walter C. Willett, Kana Wu

**Affiliations:** 1 Department of Occupational Health, Hanoi Medical University, Hanoi, Vietnam; 2 Department of Nutrition, Harvard T.H. Chan School of Public Health, Boston, Massachusetts, USA; 3 Clinical and Translational Epidemiology Unit and Division of Gastroenterology, Massachusetts General Hospital and Harvard Medical School, Boston, Massachusetts, USA; 4 Department of Epidemiology, Harvard T.H. Chan School of Public Health, Boston, Massachusetts, USA; 5 Channing Division of Network Medicine, Department of Medicine, Brigham and Women’s Hospital and Harvard Medical School, Boston, Massachusetts, USA; 6 Rally Health, San Francisco, California, USA; 7 Department of Medical Oncology, Dana-Farber Cancer Institute and Harvard Medical School, Boston, Massachusetts, USA; 8 Division of Molecular Pathological Epidemiology, Department of Pathology, Brigham and Women’s Hospital and Harvard Medical School, Boston, Massachusetts, USA; 9 Division of Cancer Epidemiology and Genetics, National Cancer Institute, Rockville, Maryland, USA

## Abstract

**Background:**

Heterocyclic amines (HCAs) in cooked meats may play a role in colorectal cancer (CRC) development.

**Objectives:**

We aimed to prospectively examine the association between estimated intakes of HCAs and meat-derived mutagenicity (MDM) in two cohorts of health professionals, the Health Professionals Follow-up Study (HPFS) and the Nurses’ Health Study (NHS).

**Methods:**

In 29,615 men and 65,875 women, intake of the HCAs 2-amino-3,8-dimethylimidazo(4,5-j)quinoxaline (MeIQx), 2-amino-1-methyl-6-phenylimidazo(4,5-b)pyridine (PhIP), 2-amino-3,4,8-trimethylimidazo(4,5-f)quinoxaline (DiMeIQx), and MDM was estimated using a 1996 cooking questionnaire, the 1994 food frequency questionnaire, and an online database. Cox proportional hazards models were used to estimate hazard ratios (HRs) and 95% confidence intervals (CIs) and to adjust for potential confounders. Estimates for both cohorts were pooled using random-effects meta-analysis.

**Results:**

Between 1996 and 2010, 418 male and 790 female CRC cases were identified. Meat mutagen intake was not statistically significantly associated with risk of CRC [highest vs. lowest quintile, pooled HR (95% CI) for MeIQx: 1.12 (0.93, 1.34), p for trend 0.23; PhIP: 1.10 (0.90, 1.33), p for trend 0.35; MDM: 1.03 (0.86, 1.24), p for trend 0.75] or subtypes of CRC defined by tumor location (proximal or distal colon, or rectum). When analyzed by source of meat, PhIP from red but not from white meat was nonsignificantly positively associated with CRC and significantly positively associated with proximal cancers [HR (95% CI) per standard deviation increase of log-transformed intake: PhIP red meat: CRC: 1.06 (0.99, 1.12), proximal: 1.11 (1.02, 1.21); PhIP white meat: CRC: 0.99 (0.94, 1.04), proximal: 1.00 (0.93, 1.09)].

**Conclusions:**

Estimated intakes of meat mutagens were not significantly associated with CRC risk over 14 years of follow-up in the NHS and HPFS cohorts. Results for PhIP from red but not from white meat warrant further investigation.

**Citation:**

Le NT, Michels FA, Song M, Zhang X, Bernstein AM, Giovannucci EL, Fuchs CS, Ogino S, Chan AT, Sinha R, Willett WC, Wu K. 2016. A prospective analysis of meat mutagens and colorectal cancer in the Nurses’ Health Study and Health Professionals Follow-up Study. Environ Health Perspect 124:1529–1536; http://dx.doi.org/10.1289/EHP238

## Introduction

Cooking meat at high temperature and for a long duration produces several mutagenic compounds such as heterocyclic amines (HCAs). In the human diet, major HCAs include 2-Amino-3,8-dimethylimidazo(4,5-*j*)quinoxaline (MeIQx), 2-amino-1-methyl-6-phenylimidazo(4,5-*b*)pyridine (PhIP), and 2-amino-3,4,8-trimethylimidazo(4,5-*f*)quinoxaline (DiMeIQx). Animal studies have provided sufficient evidence for their carcinogenic potential [Group 2B, International Agency for Research on Cancer (IARC)] of MeIQx, DiMeIQx, and PhIP (IARC 1993; NTP 2014); however, in humans, epidemiological data relating HCA intake to risk of colorectal cancer (CRC) are inconsistent (Augustsson et al. 1999; Butler et al. 2003; Cross et al. 2010; Gilsing et al. 2012; Helmus et al. 2013; Kobayashi et al. 2009; Le Marchand et al. 2002; Miller et al. 2013; Nöthlings et al. 2009; Nowell et al. 2002; Ollberding et al. 2012; Joshi et al. 2015). Given the paucity of prospective data relating HCA intake to CRC, we used data from the Nurses’ Health Study (NHS) and Health Professionals Follow-up Study (HPFS) to investigate the association between meat mutagen intake and risk of CRC. The long follow-up (14 years) and large number of cases also allowed us to assess associations by sub-sites and to conduct lagged analyses with sufficient statistical power. Our specific hypotheses were that *a*) higher intake of meat mutagens would be associated with higher risk of CRC, and *b*) associations between meat mutagens and CRC would vary by cancer sub-sites and by latency period between exposure and disease.

## Methods

### Study Population

We used data from two large prospective cohort studies: the HPFS, which included 51,529 U.S. male health professionals, 40–75 years of age at enrollment in 1986; and the NHS, which included 121,700 U.S. female nurses, 30–55 years of age at enrollment in 1976. More details on the cohorts and data collection can be found elsewhere (Colditz et al. 1997; Rimm et al. 1991). In brief, at baseline and then every 2 years thereafter, participants in both cohorts received questionnaires inquiring about medical history and lifestyle. The study protocol was approved by the Human Subjects Committee of the Brigham and Women’s Hospital and the Harvard T.H. Chan School of Public Health. Completion and return of the questionnaires was considered implied consent.

### Assessment of Diet

Dietary information was obtained from validated semi-quantitative food frequency questionnaires (FFQ) (Feskanich et al. 1993; Rimm et al. 1992; Salvini et al. 1989; Willett et al. 1985). In the HPFS, a 131-item FFQ was administered at the time the cohort was established in 1986, follow-up FFQs were mailed every 4 years (e.g., 1990, 1994, 1998). In the NHS, FFQs were administered in 1984, 1986 and then every 4 years thereafter (e.g., 1990, 1994, 1998). Because the cooking questionnaire was administered during a non-FFQ follow-up questionnaire cycle in 1996 (start of follow-up for this current study), intake of nutrients such as calcium or folate or foods such as total red meat was estimated using data from our FFQs. On each FFQ, participants were asked how often on average they ate a specific food item (using a specified portion size) during the past year. Participants were given nine categories of frequency of intake to choose from: never or < 1/month, 1–3/month, 1/week, 2–4/week, 5–6/week, 1/day, 2–3/day, 4–5/day and ≥ 6/day. We computed nutrient intake by multiplying the nutrient content of foods with the reported frequency of intake of each food from the FFQs and applied the residual method to calculate energy-adjusted nutrient intakes (Willett 2013a). Cumulative updated nutrient and diet intake was computed by averaging the intakes from all available FFQs up to the most recent 2-year follow-up cycle. We used cumulative intake to enhance our estimate of long-term dietary intake (Willett 2013b).

### Assessment of HCA Intake

To better estimate HCA intake in our cohorts, we previously conducted a pilot study (Byrne et al. 1998). In that study, we mailed a four-page cooking questionnaire inquiring about frequency of intake of 18 food/cooking method combinations as well as doneness of each item (questions were developed in collaboration with R. Sinha from the National Cancer Institute), to a random sample of 250 HPFS participants and 250 NHS participants. Cooking questionnaires were returned by 226 NHS and 216 HPFS participants and then linked to HCA levels measured in cooked meat samples (Sinha et al. 1995, 1998; Sinha and Rothman 1997) to estimate HCA intake in each cohort.

Based on the results from the pilot study we developed a cooking questionnaire, which was included in the 1996 follow-up mailing. This cooking questionnaire inquired about the frequency of intake (i.e., never, < 1/month; 1/month, 2–3/month; 1/week, 2–3/week; and ≥ 4/week) and doneness (i.e., lightly browned; medium browned; well browned; and blackened/charred) of cooked meats and fish [i.e., pan-fried, broiled, and grilled chicken; broiled fish; roast beef; pan-fried steak (NHS only); pan-fried hamburger (HPFS only); grilled or barbecued steak; and homemade beef gravy]. We used the CHARRED Database (National Cancer Institute), an online database (NCI 2006) that contains data on heterocyclic amines and meat-derived mutagenicity (MDM), a marker for overall meat mutagen exposure, which was measured using the Ames Salmonella test (Ames et al. 1975). Consumption of meat mutagens was estimated by multiplying the frequency of cooked meat intake from the cooking questionnaires with measured individual heterocyclic amine (ng/g meat) or MDM levels (revertant colonies/g meat) and standard (medium) portion size from the CHARRED Database (NCI 2006). Meat mutagen intake for bacon (HPFS and NHS) and hamburger (NHS only) was estimated using intake data from the 1994 FFQ, which is the FFQ closest to 1996. For more detail on those calculations in the HPFS and NHS, please refer to our previous publications (Wu et al. 2006, 2010).

### Case and Death Ascertainment

When a participant reported a diagnosis of CRC on the biennial questionnaire, we requested permission from the participant to obtain and review medical records. Study investigators blinded to the exposure status extracted information on stage, site, and histology of CRC (Bernstein et al. 2015; Zhang et al. 2012). We identified deaths by reviewing the National Death Index, the state vital statistics record, and death certificates that were mailed by next of kin of deceased participants with > 96% sensitivity (Rich-Edwards et al. 1994; Stampfer et al. 1984). For nonrespondents who died of CRC, we also requested permission to obtain medical records from the next of kin. After review of those medical records by study investigators we were able to confirm a diagnosis of CRC in over 98% of deceased nonrespondents who died of CRC in both cohorts.

### Exclusion Criteria

Participants were ineligible for this study if they *a*) had reported a history of any cancer (except for non-melanoma skin cancer) or ulcerative colitis before 1996, *b*) had calculated energy intake of < 800 or > 4,200 kcal/day in men, or < 600 or > 3,500 kcal/day in women, or *c*) had left the entire cooking method section on the 1996 questionnaire blank. In addition, we also excluded participants for whom HCAs/MDM could not be calculated due to missing information on bacon or hamburger intake in the 1994 FFQ or had reported information on doneness of cooked meat but not on frequency of cooked meat intake.

### Statistical Analyses

Each participant contributed person-years from the date of return of the 1996 follow-up questionnaire to the end of our follow-up period (31 January 2010 for HPFS and 30 May 2010 for NHS), date of death, or CRC diagnosis, whichever occurred first. Intakes of HCAs and MDM were divided into cohort-specific quintiles. To examine the association between quintiles of meat mutagen intake and risk of CRC, we used a Cox proportional hazards model to estimate hazard ratios (HRs) and 95% confidence intervals (CIs). In multivariable models, we adjusted for age (in months) and other known and suspected risk factors for CRC. Covariates were derived from our repeated follow-up lifestyle and medical history questionnaires and FFQs and included family history of CRC in first-degree relatives (yes vs. no), prior lower gastrointestinal endoscopy (sigmoidoscopy or colonoscopy; yes vs. no), pack-years of smoking before age 30 years (0, 0.1–4.9, 5–9.9, ≥ 10), body mass index (BMI; kg/m^2^: < 23, 23–24.9, 25–26.9, 27–29.9, ≥ 30), leisure time physical activity [metabolic equivalent (METs)–hr/week: < 3, 3–8.9, 9–17.9, 18–26.9, ≥ 27], regular aspirin or NSAID (nonsteroidal anti-inflammatory drugs) use (≥ 2 tablets/week), total caloric intake (quintiles), alcohol consumption (g/day: < 5, 5–9.9, 10–14.9, 15–29.9, ≥ 30). To assess whether observed associations may be explained by meat or fish intake per se or components of meat, we also ran additional models after including intake of total red meat, unprocessed red meat, processed meat, chicken/turkey, fish, and total saturated fat intake (all in quintiles) separately to the multivariable models. Because other dietary factors such as calcium, folate, or fiber intake have also been suggested as risk factors for CRC, we also ran models after adjusting for total calcium, total fiber, and total folate intake (in quintiles) separately. We added each dietary variable separately to the models because of possible colinearity between dietary factors. Because there is evidence suggesting a role for heme iron in colorectal carcinogenesis (heme iron can increase production of carcinogenic *N*-nitroso compounds) (Bastide et al. 2015; Cross and Sinha 2004), we also ran analyses after including heme iron intake to the multivariable models. A test for trend was calculated by treating the median of each quintile of the exposure variable as a continuous variable in the model and using a Wald test to assess statistical significance. Risk estimates from both cohorts were pooled by utilizing random-effects meta-analysis (DerSimonian and Laird 1986).

We also examined associations between meat mutagen intake and sub-sites of CRC using meat mutagen intake as a continuous variable [per standard deviation (SD) of log (natural logarithm)–transformed meat mutagen intake]. To assess whether associations may differ by risk factors for CRC, we also conducted analysis after stratification by BMI (< 25 kg/m^2^/≥ 25 kg/m^2^), smoking status (never/ever), and family history of CRC in first-degree relatives (yes/no). Tests for interaction were conducted by including cross-product terms of the stratification variable (binary) and HCAs or MDM (continuous) in the models and using a Wald test to assess statistical significance. We also examined whether frequency of meat intake cooked using a specific cooking method (proxy for temperature of cooking) such as pan-frying, broiling, grilling, barbequing, or roasting (roast beef) and outside appearance (proxy for duration of cooking; well-browned or blackened/charred vs. lightly browned or medium browned) were associated with CRC.

To examine risk of CRC 0–4 years after exposure, we limited our analyses to cases diagnosed between 1996 and 2000, and for the 4–14 year lagged analyses we limited our analyses to cases diagnosed between 2000 and 2010. A two sided *p*-value < 0.05 was considered statistically significant.

## Results

Between 1996 and 2010, we identified 418 CRC cases in men and 790 in women through follow-up of 29,615 men and 65,785 women. Lowest, median, and highest intakes of HCA and MDM in each quintile are depicted in Table S1. Median and SD of the log-transformed meat mutagen intake are presented in Table S2. Because lifestyle and dietary factors may differ by age, baseline characteristics for participants by PhIP and MDM intake were age-standardized (Table 1). Generally, participants with lower intake of PhIP and MDM were more likely to adhere to a healthier lifestyle and diet: They tended to have lower BMI and were more likely to use multivitamins and had lower intakes of total red meat, animal protein, heme iron, and alcohol, but higher intake of folate, calcium, vitamin D, and dietary fiber. In addition, participants with higher meat mutagen intake were also more likely aspirin or NSAID users (Table 1). Participants with lower MDM were also more likely to undergo lower bowel endoscopy and be more physically active. Baseline characteristics by MeIQx and DiMeIQx intake were similar to those presented for PhIP (data not shown).

**Table 1:** Age-standardized baseline characteristics of study participants in the Health Professionals Follow-up Study (*n *= 29,615) and the Nurses’ Health Study (*n *= 65,785) by PhIP and MDM intake [HCA quintiles (Q)].*^a^*

Characteristic^*b*^	HPFS				NHS			
	PhIP-Q1	PhIP-Q5	MDM-Q1	MDM-Q5	PhIP-Q1	PhIP-Q5	MDM-Q1	MDM-Q5
Number of observations	5,923	5,934	5,923	5,923	13,157	13,157	13,157	13,157
Age (years)	63.5	62.8	63.4	63.0	62.3	62.0	62.2	62.1
Body mass index (kg/m^2^)	24.9	25.9	24.9	25.9	25.6	27.3	25.4	27.4
Body mass index
< 25 kg/m^2^ (%)	56.5	40.7	56.9	41.7	59.0	45.8	61.0	44.6
≥ 25 kg/m^2^ (%)	43.5	59.3	43.1	58.3	41	54.2	39	55.4
Physical activity, MET-hr/week	34.2	34.1	34.3	33.2	19.2	16.9	20.3	15.7
Pack-years of smoking before age 30 years	10.5	11.6	10.7	11.5	7.0	7.2	7.0	7.2
Smoking status
Never (%)	48.8	43.8	49.3	43.6	47.6	41.5	46.9	43.2
Ever (%)	51.2	56.2	50.7	56.4	52.4	58.5	53.1	56.8
Family history of colorectal cancer in first-degree relative (%)	16.0	14.9	15.8	14.5	17.3	18.4	17.5	18.4
Prior lower bowel endoscopy (sigmoidoscopy or colonoscopy) (%)	28.2	27.1	29.3	24.5	18.7	17.6	19.7	17.3
Current multivitamin use (%)	57.7	52.4	56.6	51.7	56.2	50.1	57.1	50.2
Regular aspirin or NSAID use ≥ 2 tablets/week) (%)	53.2	59.2	53.0	57.7	48.9	56.4	49.1	56.1
Postmenopausal (%)	—	—	—	—	91.1	91.4	91.0	91.3
Current postmenopausal hormone use (%)^*c*^	—	—	—	—	66.8	68.2	68.9	66.2
Total red meat intake (g/day)	42.7	89.0	38.4	100.1	40.6	81.4	35.8	87.1
Unprocessed red meats (servings/day)	0.3	0.7	0.3	0.8	0.3	0.6	0.3	0.7
Processed red meats (servings/day)	0.1	0.3	0.1	0.5	0.1	0.4	0.1	0.4
Poultry (servings/day)	0.4	0.5	0.4	0.5	0.4	0.4	0.4	0.4
Fish (servings/day)	0.3	0.3	0.3	0.3	0.3	0.3	0.3	0.3
Total fruits (servings/day)	2.8	2.5	2.8	2.4	2.4	2.3	2.5	2.2
Total vegetables (servings/day)	3.2	3.3	3.2	3.2	3.0	3.2	3.1	3.1
Alcohol consumption (g/day)	2.0	2.5	2.0	2.4	4.8	7.3	5.3	6.6
Animal protein (g/day)	58.9	67.0	59.4	66.4	53.7	56.8	54.0	55.8
Vegetable protein (g/day)	28.9	25.0	28.9	24.7	20.4	18.3	20.5	18.3
Folate (μg/day)	555.7	490.6	555.7	481.1	441.3	387.1	446.8	380.7
Calcium (mg/day)	992.0	847.2	985.1	858.6	1031.1	880.6	1046.8	870.4
Vitamin D (IU/day)	472.2	412.6	478.3	403.6	379.6	319.6	383.1	315.3
Dietary fibers (g/day)	24.8	21.0	25.0	20.5	18.5	16.0	18.7	15.7
Saturated fat (g/day)	20.7	23.8	20.4	24.5	20.2	22.4	19.8	22.7
Heme iron (mg/day)	1.0	1.4	1.0	1.4	1.0	1.2	1.0	1.2
Cholesterol (mg/day)	237.0	292.8	236.6	298.6	242.3	277.4	240.4	275.9
Total calorie intake (kcal/day)	1840.1	2119.7	1802.4	2198.8	1606.0	1834.8	1590.7	1853.4
Abbreviations: MDM, meat-derived mutagenicity; MET, metabolic equivalent; PhIP, 2-amino-1-methyl-6-phenylimidazo pyridine; Q1, lowest quintile; Q5, highest quintile. ^***a***^Consumption of PhIP and MDM was estimated by multiplying the frequency of cooked meat intake from the cooking questionnaires with measured PhiP (ng/g meat) or MDM levels (revertant colonies/g meat) and standard (medium) portion size from the CHARRED Database (NCI 2006). ^***b***^Continuous variables are presented as means; all values are age-standardized except for age. ^***c***^Calculated among postmenopausal women only.								

In age-adjusted models, DiMeIQx and MDM intake was not significantly associated with risk of CRC in the HPFS (Table 2). In men, but not in women, higher intake of MeIQx was significantly associated with higher risk of CRC [highest vs. lowest quintile: HR = 1.44 (95% CI: 1.07, 1.94), *p* for trend = 0.04], but associations were attenuated after multivariable adjustment and became nonsignificant [highest vs. lowest quintile: HR = 1.22 (95% CI: 0.89, 1.68), *p* for trend = 0.35, Table 2]. From here on, “HR” denotes multivariable-adjusted HRs unless noted otherwise. Total PhIP intake was not associated with risk of CRC in multivariable models; however, when PhIP intake was investigated separately by source, results suggested a positive, albeit nonsignificant, association between PhIP from red meat in men and women as well as the pooled analysis [highest vs. lowest quintile, HR (95% CI): HPFS: 1.31 (0.93, 1.84), *p* for trend = 0.27; NHS: 1.15 (0.92, 1.45), *p* for trend = 0.14; pooled: 1.20 (0.99, 1.45), *p* for trend = 0.06; Table 2], but not from white meat. MeIQx and MDM from red meat were not significantly associated with risk of CRC [highest vs. lowest quintile, pooled HR (95% CI) for MeIQx from red meat: 1.12 (0.94, 1.35), *p* for trend = 0.21, for MDM from red meat 1.17 (0.97, 1.40), *p* for trend = 0.10] (data not shown). In addition when we examined meat mutagen intake as a continuous variable, higher intake of PhIP from red meat was nonsignificantly associated with an increased risk of CRC [pooled HR (95% CI) per SD increase of log-transformed intake: PhIP red meat: CRC: 1.06 (0.99, 1.12), *p* = 0.07, PhIP white meat: CRC: 0.99 (0.94, 1.04), *p* = 0.61 (data not shown).

**Table 2:** Hazard ratios (HR) (95% CIs) for colorectal cancer by quintiles (Q) of meat mutagen intake.

Exposure	MeIQx	PhIP total	PhIP red meat	PhIP white meat	DiMeIQx	MDM
Men (418 cases)
Age-adjusted
Q1	1.00	1.00	1.00	1.00	1.00	1.00
Q2	1.23 (0.90, 1.69)	1.13 (0.83, 1.54)	1.27 (0.91, 1.76)	1.09 (0.80, 1.47)	1.12 (0.78, 1.61)	1.13 (0.82, 1.54)
Q3	1.12 (0.81, 1.54)	1.22 (0.90, 1.65)	1.36 (0.98, 1.87)	1.25 (0.93, 1.69)	1.14 (0.87, 1.50)	0.99 (0.72, 1.37)
Q4	1.03 (0.75, 1.43)	1.33 (0.98, 1.80)	1.41 (1.02, 1.93)	1.12 (0.82, 1.52)	1.21 (0.92, 1.58)	1.30 (0.96, 1.76)
Q5	1.44 (1.07, 1.94)	1.13 (0.82, 1.57)	1.53 (1.10, 2.11)	1.17 (0.85, 1.61)	0.90 (0.67, 1.22)	1.15 (0.84, 1.57)
*p* for trend*	0.04	0.55	0.03	0.52	0.31	0.31
Multivariable adjusted^*a*^
Q1	1.00	1.00	1.00	1.00	1.00	1.00
Q2	1.16 (0.84, 1.59)	1.07 (0.78, 1.45)	1.22 (0.87, 1.70)	1.07 (0.79, 1.45)	1.12 (0.78, 1.62)	1.06 (0.77, 1.45)
Q3	1.01 (0.73, 1.40)	1.12 (0.82, 1.52)	1.26 (0.91, 1.75)	1.21 (0.89, 1.63)	1.12 (0.85, 1.47)	0.92 (0.67, 1.27)
Q4	0.92 (0.66, 1.28)	1.23 (0.90, 1.68)	1.28 (0.92, 1.77)	1.09 (0.80, 1.48)	1.14 (0.87, 1.49)	1.18 (0.86, 1.60)
Q5	1.22 (0.89, 1.68)	1.01 (0.72, 1.41)	1.31 (0.93, 1.84)	1.13 (0.82, 1.55)	0.88 (0.65, 1.19)	1.02 (0.73, 1.41)
*p* for trend*	0.35	0.97	0.27	0.66	0.24	0.84
Women (790 cases)
Age-adjusted
Q1	1.00	1.00	1.00	1.00	1.00	1.00
Q2	0.88 (0.69, 1.11)	1.19 (0.95, 1.48)	1.02 (0.81, 1.28)	1.10 (0.89, 1.35)	1.06 (0.84, 1.32)	0.84 (0.67, 1.05)
Q3	1.09 (0.88, 1.36)	1.27 (1.02, 1.58)	1.10 (0.87, 1.38)	1.05 (0.84, 1.30)	1.04 (0.83, 1.30)	0.99 (0.79, 1.23)
Q4	1.15 (0.93, 1.44)	1.25 (1.00, 1.57)	1.25 (1.00, 1.57)	1.13 (0.91, 1.41)	1.18 (0.94, 1.47)	1.14 (0.92, 1.42)
Q5	1.13 (0.91, 1.40)	1.19 (0.94, 1.49)	1.20 (0.96, 1.50)	0.91 (0.71, 1.15)	1.15 (0.93, 1.43)	1.09 (0.88, 1.35)
*p* for trend*	0.06	0.28	0.05	0.27	0.16	0.09
Multivariable adjusted^*a*^
Q1	1.00	1.00	1.00	1.00	1.00	1.00
Q2	0.86 (0.68, 1.09)	1.18 (0.94, 1.47)	1.02 (0.81, 1.28)	1.10 (0.89, 1.36)	1.06 (0.84, 1.32)	0.83 (0.66, 1.04)
Q3	1.06 (0.84, 1.32)	1.25 (1.00, 1.56)	1.08 (0.85, 1.36)	1.04 (0.83, 1.29)	1.04 (0.83, 1.30)	0.97 (0.77, 1.21)
Q4	1.11 (0.88, 1.38)	1.22 (0.97, 1.54)	1.23 (0.98, 1.54)	1.13 (0.91, 1.40)	1.18 (0.94, 1.47)	1.11 (0.89, 1.38)
Q5	1.07 (0.86, 1.34)	1.14 (0.90, 1.45)	1.15 (0.92, 1.45)	0.90 (0.71, 1.15)	1.15 (0.93, 1.43)	1.04 (0.83, 1.30)
*p* for trend*	0.19	0.49	0.14	0.26	0.16	0.23
Pooled age adjusted** (1,208 cases)
Q5 vs. Q1	1.25 (0.99, 1.57)	1.17 (0.97, 1.41)	1.31 (1.05, 1.65)	1.01 (0.79, 1.29)	1.04 (0.82, 1.32)	1.11 (0.93, 1.32)
*p* **^,#^	0.06	0.11	0.02	0.96	0.73	0.26
Pooled multivariable adjusted^a,^**^**^(1,208 cases)	
Q5 vs. Q1	1.12 (0.93, 1.34)	1.10 (0.90, 1.33)	1.20 (0.99, 1.45)	0.98 (0.80, 1.21)	1.01 (0.81, 1.24)	1.03 (0.86, 1.24)
*p* **^,#^	0.23	0.35	0.06	0.87	0.96	0.75
Q1 is the reference category. ^***a***^Adjusted for age (months), 2-year follow-up cycle, family history of colorectal cancer in first-degree relatives (yes vs. no), prior lower gastrointestinal endoscopy (sigmoidoscopy or colonoscopy; yes vs. no), pack-years of smoking before age 30 years (0, 0.1–4.9, 5–9.9, ≥ 10), body mass index (kg/m^2^: < 23, 23–24.9, 25–26.9, 27–29.9, ≥ 30), leisure time physical activity (MET-hours/week:< 3, 3–8.9, 9–17.9, 18–26.9, ≥ 27), regular aspirin or NSAID use (≥ 2 tablets/week), total caloric intake (quintiles), alcohol consumption (g/day: < 5, 5–9.9, 10–14.9, 15–29.9, ≥ 30). *Test for trend was calculated by treating the median of each quintile of the exposure variable as a continuous variable in the model and using a Wald test to assess statistical significance. **All *p* for heterogeneity > 0.05. ^*#*^*p* for trend was derived from random-effects meta-analysis.						

Associations between HCAs and CRC were similar after further adjusting for total red meat (see Table S3), calcium, fiber, folate, saturated fat, or poultry intake (data not shown). MeIQx, DiMeIQx, and MDM were not associated with CRC sub-sites, which included 155 proximal, 120 distal, and 87 rectal cases in men and 400 proximal, 216 distal, and 159 rectal cases in women (numbers do not add up to 100% of all CRC cases in each cohort because for some cases the exact sub-site at which the tumor occurred was either ambiguous or missing, and the cases were excluded from the sub-site analysis; only cases with available sub-site data were included in this analysis). However, PhIP from red but not white meat was positively associated with risk of proximal cancers [per SD of log-transformed HCAs increase: pooled HR (95% CI) = 1.11 (1.02, 1.21), *p* = 0.02, Table 3]. The association between PhIP from red meat and rectal cancer was similar to that observed for proximal cancer but did not reach statistical significance [pooled HR (95% CI) = 1.10 (0.95, 1.27), *p* = 0.22, Table 3].

**Table 3:** Pooled HR (95% CI) for CRC (per standard deviation increase of log-transformed meat mutagen intake) by sub-sites.

HCAs	Proximal colon	p*^,^**	Distal colon	p*^,^**	Rectum	p*^,^**
Cases men/women	155/400		120/216		87/159
Pooled age-adjusted
MeIQx	1.04 (0.96, 1.13)	0.33	1.02 (0.91, 1.13)	0.77	1.08 (0.95, 1.23)	0.24
PhIP total	1.07 (0.98, 1.17)	0.15	0.98 (0.89, 1.09)	0.76	1.04 (0.92, 1.19)	0.52
PhIP red meat	1.12 (1.02, 1.24)	0.02	0.98 (0.88, 1.09)	0.72	1.13 (0.98, 1.31)	0.10
PhIP white meat	1.00 (0.93, 1.09)	0.94	0.96 (0.87, 1.05)	0.35	0.97 (0.87, 1.09)	0.63
DiMeIQx	1.01 (0.92, 1.11)	0.82	1.05 (0.93, 1.18)	0.46	1.11 (0.96, 1.28)	0.14
MDM	1.04 (0.95, 1.14)	0.40	1.02 (0.92, 1.14)	0.69	1.02 (0.90, 1.15)	0.78
Pooled multivariable adjusted^*a*^
MeIQx	1.03 (0.94, 1.12)	0.52	0.97 (0.85, 1.10)	0.62	1.04 (0.91, 1.19)	0.59
PhIP total	1.07 (0.97, 1.17)	0.17	0.96 (0.86, 1.06)	0.41	1.02 (0.89, 1.16)	0.81
PhIP red meat	1.11 (1.02, 1.21)	0.02	0.94 (0.84, 1.05)	0.26	1.10 (0.95, 1.27)	0.22
PhIP white meat	1.00 (0.93, 1.09)	0.90	0.95 (0.85, 1.06)	0.33	0.96 (0.86, 1.07)	0.47
DiMeIQx	1.01 (0.92, 1.10)	0.88	1.02 (0.90, 1.16)	0.71	1.07 (0.93, 1.24)	0.34
MDM	1.03 (0.94, 1.13)	0.48	1.00 (0.89, 1.11)	0.96	0.99 (0.88, 1.12)	0.91
^***a***^Adjusted for age (months), 2-year follow-up cycle, family history of colorectal cancer in first-degree relatives (yes vs. no), prior lower gastrointestinal endoscopy (sigmoidoscopy or colonoscopy; yes vs. no), pack-years of smoking before age 30 (0, 0.1–4.9, 5–9.9, ≥ 10), body mass index (kg/m^2^: < 23, 23–24.9, 25–26.9, 27–29.9, ≥ 30), leisure time physical activity (MET-hours/week: < 3, 3–8.9, 9–17.9, 18–26.9, ≥ 27), regular aspirin or NSAID use (≥ 2 tablets/week), total caloric intake (quintiles), alcohol consumption (in g/day: < 5, 5–9.9, 10–14.9, 15–29.9, ≥ 30). *All *p* for heterogeneity > 0.05. **p-Values were derived from random-effects meta-analysis.						

Associations were similar after we separately adjusted for total red meat (see Table S4), processed red meat, unprocessed meat, chicken, fish, saturated fat, folate, calcium, or fiber intake (data not shown). Positive associations between PhIP from red meat and proximal cancers also persisted after adding heme iron intake [per SD of log-transformed HCA increase: pooled HR (95% CI): 1.12 (1.03, 1.23), *p* = 0.01] or MDM from red meat separately to the multivariable models [pooled HR (95% CI): 1.18 (1.01, 1.37), *p* = 0.04] (data not shown).

After restricting analyses to cases diagnosed within the first 4 years of follow-up we observed positive associations between intake of PhIP from red meat, but not from white meat and CRC [highest vs. lowest tertile, pooled HR (95% CI): PhIP red meat 1.39 (1.07, 1.79), *p* for trend = 0.01, PhIP white meat: 1.09 (0.74, 1.62), *p* for trend = 0.66; see Table S5]. However, results were based on a limited number of cases (255 in women and 133 in men). No association between intake of HCAs and MDM and risk of CRC were observed with longer latency periods (4–14 year lag).

In both men and women, associations between HCAs and CRC did not appear to differ by smoking status (never/ever) or family history of CRC (yes/no) with *p* for interaction > 0.05 (data not shown). There was a positive but nonsignificant association between PhIP intake and proximal cancers in overweight or obese men (BMI ≥ 25), but not in men with BMI < 25 [highest vs. lowest tertile, HR (95% CI), BMI ≥ 25: 2.35 (1.29, 4.27), BMI < 25: 0.54 (0.27, 1.10), *p* for interaction = 0.07]. The association between PhIP and proximal cancers did not differ by BMI in women [highest vs. lowest tertile, HR (95% CI), BMI ≥ 25: 1.20 (0.86, 1.69), BMI < 25: women: 0.98 (0.64, 1.49), *p* for interaction = 0.50].

No significant positive associations between cooking methods or outside appearance and risk of CRC was observed. Our results suggested a positive, albeit nonsignificant association between well-done pan-fried steak intake and CRC [data collected in women only: well-done vs. lightly browned 1.38 (95% CI: 0.89, 2.15), *p* for trend = 0.07], but results were based on a small number of cases in the reference category (*n* = 26) (see Table S6).

## Discussion

Results from these two large prospective cohort studies do not support the hypothesis that higher intake of heterocyclic amine intake per se substantially increases risk of CRC. However, we observed positive association between PhIP from red meat, but not from white meat, and proximal colon cancers. PhIP from red meat also was associated with CRC diagnosed during the first 4 years of follow-up (1996–2000), but was not associated with CRC diagnosed 4–14 years after baseline.

Although red and processed meats are considered established risk factors for CRC and the 2007 World Cancer Research Fund/American Institute for Cancer Research (WCRF/AICR) and the 2001 Continuous Update Project (CUP) reports concluded that the evidence for a link between red and processed meat and CRC is “convincing,” little is known about underlying mechanisms (WCRF/AICR 2007, 2011). Recently, the IARC Monograph Working Group classified “the consumption of processed meat as carcinogenic to humans (Group 1) based on the sufficient evidence in humans that the consumption of processed meat causes colorectal cancer” (Bouvard et al. 2015). On the other hand, the IARC Working Group classified “the consumption of red meat as probably carcinogenic based on limited evidence that the consumption of red meat causes cancers and the strong mechanistic evidence (Group 2A).” Cooking meats can result in the production of mutagens such as polycyclic aromatic hydrocarbons (PAHs) or heterocyclic amines. PAHs are produced when there is incomplete combustion, and exposure to PAHs is also possible via the environment (Cross and Sinha 2004).

Heterocyclic amines are formed when meats are cooked at high temperatures via reactions between amino acids, sugars, and creatine (Hodge 1953; Maillard 1912). More than 20 HCAs have been detected in cooked meats, and concentrations tend to increase with higher temperature and longer duration of cooking (Sugimura 1997, 2000; Turesky et al. 2007). In bacterial mutagenicity assays HCAs are mutagenic, and in animal studies both MeIQx and PhIP are very potent chemical carcinogens for multiple organs, including the colon (Kato et al. 1989; Ochiai et al. 1991; Ohgaki et al. 1986). PhIP is the most abundant HCA in the human diet, followed by MeIQx and DiMeIQx (IARC 1993). In terms of mutagenicity, however, the order is reversed, with DiMeIQx being the most and PhIP the least potent mutagen (Sinha and Rothman 1997).

Only a few studies have examined the association between meat mutagens and risk of CRC, and the results are inconsistent. For example, several U.S.-based case–control studies on HCAs and CRC observed a positive association between HCA intake and CRC or colon cancer (Butler et al. 2003; Helmus et al. 2013; Joshi et al. 2015; Le Marchand et al. 2002; Miller et al. 2013; Nowell et al. 2002), whereas a null association was observed in other studies (Augustsson et al. 1999; Gilsing et al. 2012; Kobayashi et al. 2009). Differences in exposure assessment such as type of questions on the cooking questionnaires, reference period of reported intake, and the use of different HCA databases may at least partly explain those differential findings. For example, although the U.S.-based case–control studies (Butler et al. 2003; Helmus et al. 2013; Joshi et al. 2015; Le Marchand et al. 2002; Miller et al. 2013; Nowell et al. 2002; Gilsing et al. 2012) used databases developed by Sinha and colleagues (for studies before 2006: Sinha 2002; Sinha and Rothman 1997; Sinha et al. 1995, 1998, 2001; for studies after 2006: NCI 2006), the two non-U.S. case–control studies from Sweden and Japan (Augustsson et al. 1999; Kobayashi et al. 2009) used databases accounting for cooking methods and culinary preferences in their respective populations (Skog et al. 1995; Kobayashi et al. 2009).

Two large prospective U.S.-based cohorts—the NIH-AARP Diet and Health Study (NIH-AARP) (Cross et al. 2010) and the Multiethnic Cohort Study (Ollberding et al. 2012)—have previously reported the association between HCAs per se and other meat mutagens and risk of CRC. In the NIH-AARP study, which included 2,719 CRC cases, a positive association between HCA intake and CRC was seen in the NIH-AARP study after 7 years of follow-up (Cross et al. 2010). In that study, higher intake of DiMeIQx, MeIQx, and MDM but not PhIP was associated with higher risk of colon cancers, but no associations were observed for rectal cancers. In the Multiethnic Cohort Study (MEC), HCA intake was not associated with CRC (*n* = 1,757 cases) regardless of sub-sites (colon vs. rectum) after 15 years of follow-up (Ollberding et al. 2012). It is unclear why associations in the NIH-AARP differ from those observed in the MEC and our study. All four cohorts used the CHARRED Database to estimate meat mutagen intake, and the age distribution of the study population at the time cooking questionnaires were administered did not vary considerably between those studies (NHS and HPFS in 1996, 50–75 years; MEC, 45–75 years; NIH-AARP, 50–71 years).

However, follow-up time in the NIH-AARP was shorter (7 years) than those in the other studies (MEC, 15 years; NHS and HPFS, 14 years). Furthermore, the majority of participants in the HPFS, NHS, and the NIH-AARP were white (> 90%) (Cross et al. 2010), whereas the MEC study consisted of a more ethnically diverse population and included Japanese-Americans (29.5%), whites (26%), Latinos (21.3%), African Americans (15.9%), and Native Hawaiians (7.3%) (Ollberding et al. 2012). In addition, HPFS, NHS, and NIH-AARP participants were on average better educated than MEC participants (Kolonel et al. 2000; Schatzkin et al. 2001). Multivariable models in the three cohorts included a similar set of covariates consisting of known and suspected risk factors for CRC. However, although the NIH-AARP cohort and MEC cohort both adjusted for calcium and fiber intake, the final model in the MEC study also included folate and vitamin D intake. In our study, after including calcium, fiber, or folate intake separately to our final multivariable models, results were similar.

When analyzed by cancer sub-sites, PhIP from red meat, but not from white meat, was positively associated with proximal cancers, and associations remained similar after adjusting for red or processed meat intake. These findings are in agreement with those of a previous case–control study by Helmus et al. (2013), which also used the CHARRED Database to estimate meat mutagen intake; the authors observed positive associations between meat mutagen intake from red meat, but not from white meat and risk of CRC. However, in a multi-center case–control study by Kampman et al. (1999) a nonsignificant positive association between meat mutagen index (calculated by multiplying frequency of meat intake with doneness of meat intake) from white, but not red meat, and risk of colon cancer was observed in men, but not in women. In women, mutagen index from white or red meat was not associated with risk of colon cancer. Because the study by Kampman et al. (1999) did not estimate meat mutagen intake per se, it is not possible to compare the results between those two case–control studies.

It is unclear why PhIP from red but not from white meat is positively associated with risk of CRC in our study. Differential findings by source of meat suggest that PhIP intake by itself is likely not associated with CRC. It possible that PhIP from red meat may be a marker for some unknown mutagenic compound(s) related specifically to cooking of red meat or some compounds in red meat. Evidence for the presence of other previously unknown mutagens in cooked red meat comes from a recent study, which identified a novel HCA—2-amino-1,7-dimethylimidazo[4,5-g]quinoxaline (7-MeIgQx)—in cooked beef. In grilled beef patties the concentration of 7-MeIgQx was comparable with that of PhIP or even higher (depending on the cooking temperature) (Turesky et al. 2007). The CHARRED Database does not provide data on 7-MeIgQx. However, in our study, results for PhIP from red meat and proximal cancers were similar after adding intake of total red meat, heme iron, or MDM from red meat (a marker for total mutagenic activity) to our multivariable models.

HCAs are mutagens and can form DNA adducts, but their potential role, if any, at different stages (i.e., during earlier vs. later stages) during colorectal carcinogenesis remains unknown (Cross and Sinha 2004). In our study, positive associations for PhIP from red meat were restricted to cases diagnosed within the first 4 years of follow-up (1996–2000). Interestingly, adenoma studies have generally observed positive associations between meat mutagen intake and risk of adenoma or adenoma recurrence, particularly distal adenoma, even though findings relating meat mutagens to stage or size of adenoma are inconsistent (Martinez et al. 2007; Rohrmann et al. 2009; Sinha et al. 2005). Reverse causation resulting from changes in diet due to symptoms related to undiagnosed CRC may also explain these findings. However, we would expect that symptomatic participants would decrease rather than increase their intake of well-done/charred red meat. Our results were based on a limited number of cases and we cannot exclude the possibility that the aforementioned findings are due to chance.

Other possible mechanisms through which red meat consumption can increase risk of CRC include heme iron (Bastide et al. 2015), which is higher in red than in white meat, and may also facilitate endogenous nitrosation (i.e., the production of carcinogenic *N*-nitroso compounds) (Cross and Sinha 2004). In a previous study from our group using data from the NHS and HPFS in women, our findings were suggestive of a positive, albeit nonsignificant association between heme iron intake and CRC [highest vs. lowest quintile: HR = 1.21 (95% CI: 0.96, 1.52) *p* for trend = 0.10]. In men, however, heme iron intake was not associated with risk of CRC (Zhang et al. 2011).

The strengths of our study include its prospective design, the large sample size and long follow-up of 14 years, as well as the availability of detailed and repeated assessment of diet and lifestyle factors through our follow-up questionnaires, which enabled us to adjust for a variety of potential confounders and examine associations by sub-sites as well as conduct latency analyses. Although we were able to detect positive associations, we cannot exclude the possibility of misclassification of exposure. Nondifferential misclassification of exposure (e.g., highest vs. lowest quintile of meat mutagen intake) generally attenuates associations toward the null, which may at least partly explain our null findings. For example, our results were based on a one-time assessment of HCA intake, and we were not able to account for factors that can change HCA content in cooked meats, such as frequent flipping of meat during grilling or frying (Tran et al. 2002), use of marinades (Salmon et al. 1997), microwaving meats before cooking (Felton et al. 1994), or removing the charred portion of meat before eating.

Furthermore, HCAs by themselves are not carcinogenic and require metabolic activation via xenobiotic metabolizing enzymes (XME) (Boobis et al. 1994; Chou et al. 1995; Minchin et al. 1992). However, we did not assess associations by genetic susceptibility. Only a few epidemiological studies have examined interactions between HCA intakes per se and single-nucleotide polymorphisms in XME with regard to risk of CRC, and findings are inconclusive (Butler et al. 2008; Gilsing et al. 2012; Kobayashi et al. 2009; Nöthlings et al. 2009). Another source for potential misclassification is that HCA intake was estimated based on a limited number of questions. However, cooking method questions were based on a previous pilot study, which established the set of questions to enhance estimation of HCA intake in our cohorts (Byrne et al. 1998). Further, as with every observational study we can never exclude the possibility of residual confounding.

In conclusion, estimated intakes of meat mutagens were not significantly associated with CRC risk over 14 years of follow-up in the NHS and HPFS cohorts. Results for PhIP from red but not from white meat warrant further investigation.

## Supplemental Material

(203 KB) PDF
