# Supplementary material for: A Prospective Analysis of Meat Mutagens and Colorectal Cancer in the Nurses’ Health Study and Health Professionals Follow-up Study
Source: Environ Health Perspect. 2016 Apr 22;124(10):1529–36. doi: 10.1289/EHP238 (PMC5047780; doi:10.1289/EHP238)
Supplement: (203 KB) PDF [file EHP238.s001.acco.pdf]

DOI: 10.1289/EHP238

Note to readers with disabilities: *EHP* strives to ensure that all journal content is accessible to all readers. However, some figures and Supplemental Material published in *EHP* articles may not conform to 508 standards due to the complexity of the information being presented. If you need assistance accessing journal content, please contact ehp508@niehs.nih.gov. Our staff will work with you to assess and meet your accessibility needs within 3 working days.

Supplemental Material

A Prospective Analysis of Meat Mutagens and Colorectal Cancer in the Nurses' Health Study and Health Professional Follow-up Study

Ngoan Tran Le, Fernanda Alessandra Silva Michels, Mingyang Song, Xuehong Zhang, Adam M. Bernstein, Edward L. Giovannucci, Charles S. Fuchs, Shuji Ogino, Andrew T. Chan, Rashmi Sinha, Walter C. Willett, and Kana Wu

Table of Contents

Table S1. Median (lowest, highest) estimated meat mutagen intake by quintiles in men and women.

Table S2. Median and standard deviation (SD) of log-transformed (natural logarithm) meat mutagen intake in men and women.

Table S3. Multivariable hazard ratios (HR) and 95% confidence intervals (95% CI) for meat mutagen intake and colorectal cancer in men and women-1996-2010 after adjustment for meat intake.

Table S4. Pooled multivariable hazard ratios (HR) and 95% confidence intervals (95% CI) for meat mutagens and colorectal cancer sub-sites (per standard deviation increase of log-transformed meat mutagen intake) -1996-2010 after adjustment for meat intake.

Table S5. Multivariable hazard ratios (HR) and 95% confidence intervals for lagged analysis of intake of meat mutagens and risk of colorectal cancer.

Table S6. Pooled multivariable adjusted pooled HR and 95%CI for colorectal cancer by cooking method and outside appearance (doneness).

Table S1. Median (lowest, highest) estimated meat mutagen intake by quintiles in men and women.

Quintiles	Q1<br>Median<br>(lowest, highest)	Q2<br>Median<br>(lowest, highest)	Q3<br>Median<br>(lowest, highest)	Q4<br>Median<br>(lowest, highest)	Q5<br>Median<br>(lowest, highest)
Men					
MeIQx	1.7 (0, 4.2)	7.7 (4.1, 13.6)	29.1 (13.3, 36.2)	46.5 (35.6, 64.9)	89.1 (63.5, 353.2)
PhIP total	17.7 (0, 33.5)	47.1 (31.7, 63.9)	80.3 (61.1, 105.4)	129.0 (100.9, 172.7)	234.1 (165.4, 2495.6)
PhIP red	0 (0, 7.9)	8.7 (8.7, 17.6)	21.7 (17.6, 31.4)	44.0 (31.4, 55.3)	85.7 (55.7, 2015.9)
PhIP white	5.6 (0, 17.1)	23.1 (14.5, 33.1)	43.2 (29.9, 60.0)	87.3 (56.9, 113.7)	172.8 (110.5, 1049.8)
DiMeIQx	0 (0, 0)	0.2 (0.1, 0.2)	0.6 (0.2, 0.9)	1.2 (0.9, 2.2)	4.0 (1.9, 39)
MDM	837 (0, 1755.3)	2536.8 (1704.5, 3362.6)	4143.8 (3337.7, 5114.0)	6178.1 (5092.9, 7751.4)	10199.8 (7717.6, 54285.3)
Women					
MeIQx	2.0 (0, 5.7)	16.9 (5.7, 35.3)	66.7 (34.8, 82.5)	108.3 (80.7, 146.0)	206.7 (143.8, 970.2)
PhIP total	17.7 (0, 35.6)	60.6 (35.3, 92.6)	114.9 (92.0, 146.0)	180.6 (145.1, 223.2)	295.2 (222.9, 1665.7)
PhIP red	0 (0, 8.1)	12.2 (8.7, 23.1)	51.6 (23.6, 85.0)	97.9 (85.5, 149.1)	188.9 (145.1, 1460.5)
PhIP white	4.1 (0, 14.2.0)	20.4 (13.9, 27.7)	36.7 (27.2, 50.9)	74.4 (49.4, 98.0)	146.4 (95.5, 1508.4)
DiMeIQx	0 (0, 0.6)	3.9 (0.6, 5.1)	8.3 (5.1, 11.0)	13.6 (10.7, 17.7)	24.9 (17.4, 120.0)
MDM	965.2 (0, 2408.6)	3822.8 (2392.1, 5333.6)	6812.4 (5303.8, 8556.1)	10447.0 (8481.1, 13129.5)	17571.9 (12981.3, 171620.4)

Table S2. Median and standard deviation (SD) of log-transformed (natural logarithm) meat mutagen intake in men and women.

Log Transformed[a]	Men Median (SD)	Women Median (SD)
MeIQx	3.4 (2.3)	4.2 (2.3)
PhIP total	4.4 (2.0)	4.7 (1.9)
PhIP red	3.1 (3.9)	4.0 (3.9)
PhIP white	3.8 (2.5)	3.6 (2.6)
DiMeIQx	-0.5 (3.4)	2.1 (2.9)
MDM	8.3 (2.1)	8.8 (2.1)

[a]Standard deviation of log transformed meat mutagen intake were used to examine associations between meat mutagen intake and sub-sites of CRC using meat mutagen intake as a continuous variable.

Table S3 -Multivariable hazard ratios (HR) and 95% confidence intervals (95% CI) for meat mutagen intake and colorectal cancer in men and women-1996-2010 after adjustment for meat intake.

	MeIQx	PhIP total	PhIP red	PhIP white	DiMeIQx	MDM
Men (418 cases)[a]						
Q1	1.00	1.00	1.00	1.00	1.00	1.00
Q2	1.11 (0.80, 1.54)	1.03 (0.75, 1.41)	1.20 (0.85, 1.69)	1.09 (0.80, 1.47)	1.11 (0.77, 1.60)	1.02 (0.74, 1.40)
Q3	0.92 (0.65, 1.31)	1.05 (0.77, 1.45)	1.21 (0.86, 1.72)	1.22 (0.90, 1.65)	1.09 (0.83, 1.44)	0.86 (0.62, 1.21)
Q4	0.80 (0.55, 1.15)	1.16 (0.84, 1.59)	1.18 (0.82, 1.69)	1.10 (0.81, 1.51)	1.09 (0.82, 1.43)	1.06 (0.76, 1.47)
Q5	1.02 (0.71, 1.47)	0.95 (0.67, 1.34)	1.19 (0.82, 1.73)	1.14 (0.82, 1.58)	0.85 (0.62, 1.15)	0.90 (0.64, 1.28)
P for trend[*]	0.94	0.71	0.74	0.66	0.17	0.61
Women (790 cases)[a]						
Q1	1.00	1.00	1.00	1.00	1.00	1.00
Q2	0.88 (0.69, 1.11)	1.19 (0.95, 1.49)	1.04 (0.82, 1.31)	1.11 (0.89, 1.37)	1.06 (0.84, 1.33)	0.84 (0.67, 1.06)
Q3	1.08 (0.85, 1.36)	1.26 (1.01, 1.58)	1.11 (0.87, 1.40)	1.05 (0.84, 1.31)	1.03 (0.81, 1.30)	0.98 (0.78, 1.23)
Q4	1.12 (0.88, 1.42)	1.23 (0.97, 1.55)	1.25 (0.99, 1.59)	1.15 (0.93, 1.44)	1.13 (0.89, 1.44)	1.11 (0.88, 1.40)
Q5	1.04 (0.81, 1.33)	1.11 (0.86, 1.42)	1.14 (0.89, 1.46)	0.94 (0.74, 1.20)	1.06 (0.83, 1.35)	1.00 (0.79, 1.27)
P for trend[*]	0.43	0.80	0.31	0.42	0.71	0.49
Pooled(1,208 cases)[a]						
Q5 vs. Q1	1.03 (0.84, 1.27)	1.05 (0.86, 1.29)	1.15 (0.94, 1.42)	1.01 (0.83, 1.22)	0.97 (0.78, 1.20)	0.97 (0.80, 1.18)
P[**,#]	0.75	0.63	0.18	0.96	0.76	0.76

[a]Adjusted for age (months), 2-year follow-up cycle, family history of colorectal cancer in first degree relative(yes vs. no), prior lower gastrointestinal endoscopy (sigmoidoscopy or colonoscopy, yes vs. no), pack-years of smoking before age 30(0, 0.1-4.9, 5-9.9, ≥10),

body mass index (in kg/m2: <23, 23-24.9, 25-26.9, 27-29.9, ≥30), physical activity (in metabolic equivalent-hours/week:<3, 3-8.9, 9-17.9, 18-26.9, ≥27), regular aspirin or NSAID use (≥2 tablets/week), total caloric intake (quintiles), alcohol consumption (in g/d:<5, 5-9.9, 10-14.9, 15-29.9, ≥30) and total red meat (quintiles) except for HRs for PhIP white which were adjusted for chicken/turkey (quintiles).

[*] Test for trend was calculated by treating the median of each quintile of the exposure variable as a continuous variable in the model and using a Wald-test to assess statistical significance.

[**] *P* for trend was derived from random-effects meta-analysis.

[#] All *P* for heterogeneity >0.05.

Table S4. Pooled multivariable hazard ratios (HR) and 95% confidence intervals (95% CI) for meat mutagens and colorectal cancer sub-sites (per standard deviation increase of log-transformed meat mutagen intake) -1996-2010 after adjustment for meat intake.

HCAs	Proximal colon HR (95%CI)	$P^{*,**}$	Distal colon HR (95%CI)	$P^{*,**}$	Rectum HR (95%CI)	$P^{*,**}$
Cases men/women	155/400		120/216		87/159	
Pooled[a]						
MeIQx	1.03 (0.94, 1.13)	0.57	0.93 (0.75, 1.14)	0.46	0.99 (0.85, 1.14)	0.85
PhIP total	1.07 (0.97, 1.17)	0.20	0.94 (0.84, 1.05)	0.30	0.99 (0.87, 1.13)	0.90
PhIP Red	1.13 (1.01, 1.27)	0.03	0.91 (0.81, 1.03)	0.13	1.06 (0.91, 1.22)	0.47
PhIP white	1.01 (0.93, 1.10)	0.73	0.95 (0.86, 1.05)	0.30	0.98 (0.87, 1.10)	0.74
DiMeIQx	1.00 (0.91, 1.10)	0.99	1.01 (0.89, 1.15)	0.87	1.05 (0.90, 1.22)	0.54
MDM	1.03 (0.94, 1.13)	0.53	0.99 (0.88, 1.10)	0.82	0.96 (0.85, 1.09)	0.55

[a] Adjusted for age (months), 2-year follow-up cycle, family history of colorectal cancer in first degree relative (yes vs. no), prior lower gastrointestinal endoscopy (sigmoidoscopy or colonoscopy, yes vs. no), pack-years of smoking before age 30 (0, 0.1-4.9, 5-9.9, ≥10), body mass index (in kg/m2: <23, 23-24.9, 25-26.9, 27-29.9, ≥30), physical activity (in metabolic equivalent of task-hours/week:<3, 3-8.9, 9-17.9, 18-26.9, ≥27), regular aspirin or NSAID use (≥2 tablets/week), total caloric intake (quintiles), alcohol consumption (in g/d:<5, 5-9.9, 10-14.9, 15-29.9, ≥30) and total red meat (quintiles) except for the PhIP white adjusted for chicken/turkey (quintiles).

[*] *P*-values were derived from random-effects meta-analysis.

[**] All *P* for heterogeneity >0.05.

Table S5. Multivariable hazard ratios (HR) and 95% confidence intervals for lagged analysis of intake of meat mutagens and risk of colorectal cancer.

	MeIQx	PhIP total	PhIP red	PhIP white	DiMeIQx	MDM
1996-2000 (0-4 year lag)						
NHS 255 cases [a]						
T1	1.00	1.00	1.00	1.00	1.00	1.00
T2	1.04 (0.76, 1.42)	1.21 (0.89, 1.66)	0.96 (0.70, 1.33)	1.19 (0.88, 1.61)	1.22 (0.89, 1.65)	1.07 (0.79, 1.46)
T3	1.17 (0.86, 1.59)	1.40 (1.02, 1.92)	1.32 (0.97, 1.79)	1.29 (0.94, 1.76)	1.14 (0.83, 1.57)	1.14 (0.83, 1.56)
P for trend*	0.25	0.04	0.10	0.24	0.61	0.28
HPFS 133 cases [a]						
T1	1.00	1.00	1.00	1.00	1.00	1.00
T2	0.77 (0.47, 1.24)	1.47 (0.94, 2.27)	1.31 (0.82, 2.08)	1.06 (0.70, 1.62)	1.38 (0.89, 2.14)	0.84 (0.53, 1.30)
T3	1.02 (0.65, 1.61)	1.24 (0.77, 1.98)	1.57 (0.98, 2.50)	0.86 (0.54, 1.37)	1.14 (0.71, 1.83)	0.93 (0.58, 1.49)
P for trend*	0.26	0.62	0.24	0.69	0.99	0.86
NHS and HPFS Pooled 388 cases[a]						
T3 vs. T1	1.12 (0.87, 1.45)	1.34 (1.03, 1.75)	1.39 (1.07, 1.79)	1.09 (0.74, 1.62)	1.14 (0.88, 1.48)	1.07 (0.83, 1.39)
P[**,#]	0.38	0.03	0.01	0.66	0.33	0.61
2000-2010 (4-14 year lag)						
NHS 535 cases [a]						
T1	1.00	1.00	1.00	1.00	1.00	1.00
T2	1.02 (0.82, 1.27)	1.18 (0.96, 1.45)	1.02 (0.82, 1.26)	1.06 (0.86, 1.29)	0.96 (0.78, 1.19)	1.09 (0.88, 1.35)
T3	1.14 (0.92, 1.41)	1.01 (0.81, 1.27)	1.12 (0.91, 1.39)	0.88 (0.71, 1.10)	1.09 (0.88, 1.35)	1.15 (0.93, 1.43)

P for trend[*]	0.44	0.51	0.55	0.02	0.48	0.51
HPFS 285 cases [a]						
T1	1.00	1.00	1.00	1.00	1.00	1.00
T2	1.02 (0.75, 1.38)	1.04 (0.78, 1.38)	0.96 (0.72, 1.29)	1.06 (0.80, 1.41)	1.01 (0.76, 1.34)	0.91 (0.68, 1.22)
T3	0.99 (0.73, 1.34)	0.95 (0.70, 1.30)	1.00 (0.74, 1.36)	1.09 (0.81, 1.47)	0.97 (0.72, 1.30)	0.95 (0.70, 1.28)
P for trend[*]	0.90	0.93	0.93	0.41	0.22	0.94
NHS and HPFS						
Pooled 820 cases[a]						
T3 vs. T1	1.08 (0.91, 1.29)	0.99 (0.83, 1.19)	1.08 (0.91, 1.29)	0.96 (0.78, 1.18)	1.05 (0.88, 1.24)	1.08 (0.90, 1.29)
P[**,#]	0.37	0.93	0.38	0.68	0.61	0.42

[a]Adjusted for age (months), 2-year follow-up cycle, family history of colorectal cancer in first degree relative(yes vs. no), prior lower gastrointestinal endoscopy (sigmoidoscopy or colonoscopy, yes vs. no), pack-years of smoking before age 30 (0, 0.1-4.9, 5-9.9, ≥10), body mass index (in kg/m2: <23, 23-24.9, 25-26.9, 27-29.9, ≥30), physical activity (in metabolic equivalent of task-hours/week:<3, 3-8.9, 9-17.9, 18-26.9, ≥27), regular aspirin or NSAID use (≥2 tablets/week), total caloric intake (quintiles), alcohol consumption (in g/d:<5, 5-9.9, 10-14.9, 15-29.9, ≥30).

[*]Test for trend was calculated by treating the median of each quintile of the exposure variable as a continuous variable in the model and using a Wald-test to assess statistical significance.

[**]All *P* for heterogeneity >0.05.

[#] *P* for trend was derived from random-effects meta-analysis.

Table S6 - Pooled multivariable adjusted pooled HR and 95%CI for colorectal cancer by cooking method and outside appearance (doneness).

Meat items		Reference T1	Highest exposure T3	Cases T1/T3	T3 vs. T1 HR (95%CI) [a]	P [*,**]
Pan-fried chicken	F	Never	1/mo +	569/301	1.00 (0.86, 1.15)	0.96
	O	Lightly browned	Well browned/charred	111/55	1.01 (0.72, 1.44)	0.94
Broiled chicken	F	<1/mo	1/wk +	267/478	0.93 (0.80, 1.09)	0.38
	O	Lightly browned	Well browned/charred	228/100	0.91 (0.71, 1.16)	0.43
Barbequed chicken	F	<1/mo	1/wk +	292/513	0.98 (0.84, 1.13)	0.75
	O	Lightly browned	Well browned/charred	103/118	0.99 (0.75, 1.31)	0.96
Broiled fish	F	<1/mo	1/wk +	234/608	0.89 (0.77, 1.04)	0.15
	O	Medium browned	Blackened/charred	499/104	1.10 (0.88, 1.37)	0.39
Roast beef	F	<1/mo	2/wk +	96/798	1.14 (0.91, 1.41)	0.25
	O	Lightly browned	Well browned/charred	184/350	1.13 (0.94, 1.36)	0.18
Barbequed steak	F	<1/mo	1/wk +	330/514	1.13 (0.97, 1.30)	0.11
	O	Lightly browned	Well browned/charred	90/320	1.08 (0.85, 1.37)	0.52
Beef gravy	F	Never	1/mo+	454/367	1.11 (0.97, 1.29)	0.14
	O	Lightly browned	Well browned/charred	126/190	0.98 (0.70, 1.37)	0.92
Pan-fried hamburger[b]	F	< 1/mo	2/mo +	129/205	1.02 (0.81, 1.28)	0.89
	O	Lightly browned	Well browned/charred	18/139	1.44 (0.87, 2.38)	0.15
Pan-fried steak[c]	F	< 1/mo	2/mo +	525/140	1.23 (1.01, 1.28)	0.20
	O	Lightly browned	Well browned/charred	26/101	1.38 (0.89, 2.15)	0.07

[a]Adjusted for age (months), 2-year follow-up cycle, family history of colorectal cancer in first degree relative (yes vs. no), prior lower gastrointestinal endoscopy (sigmoidoscopy or colonoscopy, yes vs. no), pack-years of smoking before age 30 (0, 0.1-4.9, 5-9.9, ≥10), body mass index (in kg/m2: <23, 23-24.9, 25-26.9, 27-29.9, ≥30), physical activity (in metabolic equivalent of task-hours/week: <3,

3-8.9, 9-17.9, 18-26.9, ≥27), regular aspirin or NSAID use (≥2 tablets/week), total caloric intake (quintiles), alcohol consumption (in g/d: <5, 5-9.9, 10-14.9, 15-29.9, ≥30);

[*]All *P* for heterogeneity >0.05;

[**] *P* for trend was derived from random-effects meta-analysis;

[b] Collected in HPFS only;

[c] collected in NHS only;

F: Frequency of intake;

O: Outside appearance (doneness).
